# Carbohydrate-active enzymes from pigmented *Bacilli*: a genomic approach to assess carbohydrate utilization and degradation

**DOI:** 10.1186/1471-2180-11-198

**Published:** 2011-09-05

**Authors:** Nicola Manzo, Enrica D'Apuzzo, Pedro M Coutinho, Simon M Cutting, Bernard Henrissat, Ezio Ricca

**Affiliations:** 1 Department of Structural and Functional Biology, Federico II University of Naples, MSA, via Cinthia 4, 80126 Napoli, Italy; 2 Architecture et Fonction des Macromolécules Biologiques, UMR6098, CNRS and Universités d'Aix-Marseille I & II, Case 932, 163 Av de Luminy, 13288 Marseille cedex 9, France; 3 School of Biological Sciences, Royal Holloway University of London, Egham, Surrey TW20 0EX, UK

## Abstract

**Background:**

Spore-forming *Bacilli *are Gram-positive bacteria commonly found in a variety of natural habitats, including soil, water and the gastro-intestinal (GI)-tract of animals. Isolates of various *Bacillus *species produce pigments, mostly carotenoids, with a putative protective role against UV irradiation and oxygen-reactive forms.

**Results:**

We report the annotation of carbohydrate active enzymes (CAZymes) of two pigmented *Bacilli *isolated from the human GI-tract and belonging to the *Bacillus indicus *and *B. firmus *species. A high number of glycoside hydrolases (GHs) and carbohydrate binding modules (CBMs) were found in both isolates. A detailed analysis of CAZyme families, was performed and supported by growth data. Carbohydrates able to support growth as the sole carbon source negatively effected carotenoid formation in rich medium, suggesting that a catabolite repression-like mechanism controls carotenoid biosynthesis in both *Bacilli*. Experimental results on biofilm formation confirmed genomic data on the potentials of *B. indicus *HU36 to produce a levan-based biofilm, while mucin-binding and -degradation experiments supported genomic data suggesting the ability of both *Bacilli *to degrade mammalian glycans.

**Conclusions:**

CAZy analyses of the genomes of the two pigmented *Bacilli*, compared to other *Bacillus *species and validated by experimental data on carbohydrate utilization, biofilm formation and mucin degradation, suggests that the two pigmented *Bacilli *are adapted to the intestinal environment and are suited to grow in and colonize the human gut.

## Background

Spore-forming *Bacilli *are aerobic, Gram positive organisms sharing a common attribute of being able to differentiate into an endospore (spore), a quiescent cell form characterized by several protective layers surrounding a dehydrated cytoplasm [1]. This structural organization makes the spores extremely resistant to external physical and chemical insults and able to survive almost indefinitely in the absence of water and nutrients [1].

The soil is generally indicated as the main habitat of aerobic spore-formers, however, spores have been found in diverse environments including rocks, dust, aquatic environments, and the gut of various insects and animals [2]. Recent reports have highlighted the fact that large numbers of aerobic spore-formers can be isolated from fecal and intestinal samples of healthy animals [3,4], including humans [5,6]. Hong and colleagues [2] have reported that an average of 10^4 ^colony forming units (CFU) of aerobic spore-formers are isolated from human feces collected in different countries and from people with different dietary habits. These observations, together with a series of reports indicating that *B. subtilis*, the model system for spore-formers, can conduct its entire life cycle in the animal gut [7,8], have suggested the hypothesis that the gut is the real habitat of spore-formers [9]. These spore-forming bacteria would enter the mammalian GI-tract in the spore form, safely transit across the stomach, germinate and grow in the upper part of the small intestine, sporulate in the lower part of the intestine and finally be excreted in the spore form [9].

It has long been known that some aerobic *Bacilli *are pigmented and examples include strains of *B. megaterium *[10], *B. atrophaeus *[11], *B. indicus *[12], *B. cibi *[13], *B. vedderi *[14], *B. jeotgali *[15], *B. okuhidensis *[16], *B. clarkii *[17], *B. pseudofirmus *[17] and *B. firmus *[18]. More recently, a large number of pigmented *Bacilli *have been isolated and their pigments identified as carotenoids [19]. Those carotenoids, found associated with either vegetative cells or spores [20], are thought to provide resistance to UV irradiation and reactive oxygen species. For their strong antioxidant activity carotenoids of plant, microbial or synthetic origin have several potential applications in the cosmetic, pharmaceutical and food industries. For example, carotenoids have been proposed to prevent the onset of chronic diseases [21] and reduce cancer-risk [22] in humans and, also for this reason, are widely marketed as dietary supplements. Non-pathogenic bacteria, able to colonize the human gut and able to produce carotenoids are, therefore, particularly desirable as food supplements and/or functional food ingredients.

Two pigmented *Bacilli, B. firmus *GB1 and *B. indicus *HU36, producing pink and yellow/orange carotenoids, respectively [19], have been characterized in detail and their genomes completely sequenced (Sequence files downloadable from http://www.agf.liv.ac.uk:8088/454/Bacillus_Download/200909/30/. Both strains have been isolated from human intestinal samples [6,8] and have been proposed as probiotic strains [19,20].

Here we report the annotation of the carbohydrate active enzymes (CAZymes) of *B. firmus *GB1 and *B. indicus *HU36. CAZymes are enzymes involved in the synthesis and degradation of carbohydrates that, for the great variability of their substrates, comprise an extremely vast family of proteins. CAZymes are organized by the CAZy database http://www.cazy.org into five main classes: i) glycoside hydrolases (GH), comprising glycosidases and transglycosylases [23], ii) glycosyl transferases (GT), that catalyse the formation of glycosidic bonds between phospho-activated sugar residues and an acceptor such as a polysaccharide, a lipid or a protein [24], iii) polysaccharide lyases (PL) that eliminate activated glycosidic linkages present in acidic polysaccharides [25], iv) carbohydrate esterases (CE), that remove ester-based modifications [25], and v) carbohydrate binding modules (CBM), non-catalytic protein domains [26]. Each of those classes are then sub-divided into several families, that group together enzymes on the base of structural and functional properties. The number and type of CAZymes carried by an organism has been used as a marker to assess the adaptation of that organism to a specific environment. Examples are species of the *Bacteroides *genus [27] and the Archaeon *Methanobrevibacter smithii *[28] identified as adapted to the human gut mainly based on their CAZy profile.

## Results and discussion

### *B. indicus *HU36 and *B. firmus *GB1 genomes contain high numbers of CAZymes

Putative CAZymes in *B. firmus *GB1 and *B. indicus *HU36 were identified using the CAZy annotation pipeline (Additional Files 1 and 2, respectively) and compared to those of a selection of spore-forming *Bacilli *(Table 1). A total of 140 and 119 CAZymes were identified in the *B. firmus *and *B. indicus *genomes, respectively. This value is similar to the number of CAZymes found in strains of the *B. subtilis/B. amyloliquefaciens *group, all having a total number of CAZymes ranging between 115 and 145 (Table 1). A lower total number of CAZymes was found in the other spore-forming species considered in this study (Table 1). Among the analyzed species, thermophilic strains of *Geobacillus *and *Alicyclobacillus *and the facultative alkaliphile strain of *B. pseudofirmus *showed a total number of CAZymes significantly lower than the other *Bacilli *(Table 1). A comparison of the five CAZyme classes mostly confirmed the results obtained analyzing the total number of CAZymes. In particular, like strains of the *B. subtilis/B. amyloliquefaciens *group, *B. indicus *and *B. firmus *showed a high number of glycoside hydrolases (GH) and carbohydrate binding modules (CBM) and average numbers of glycosyl transferases (GT), polysaccharide lyases (PL) and carbohydrate esterases (CE) (Table 1).

**Table 1:** Comparative analysis of the number of putative genes for the five CAZyme categories in selected spore-forming *Bacilli*

Species	GH^a^	GT^b^	PL^c^	CE^d^	CBM^e^	Total
***Bacillus firmus *GB1**	**58**	**42**	**2**	**14**	**24**	**140**
***Bacillus indicus *HU36**	**33**	**48**	**0**	**11**	**27**	**119**
*Bacillus clausii *KSM-K16	43	30	4	14	11	102
*Bacillus cereus *ATCC14579	28	48	0	15	13	104
*Bacillus cereus *ATCC10987	20	42	0	17	14	93
*Bacillus cereus *AH187	26	40	0	18	16	100
*Bacillus cereus *G9842	28	48	0	18	15	109
*Bacillus pumilus *SAFR-032	35	34	2	19	4	94
*Bacillus subtilis *subsp. spizizenii str.W23	42	37	6	13	27	125
*Bacillus subtilis *subsp. natto BEST195	55	38	5	13	34	145
*Bacillus subtilis *subsp. subtilis str.168	48	40	6	13	24	131
*Bacillus amyloliquefaciens *DSM7	41	36	3	10	25	115
*Bacillus pseudofirmus *OF4	22	22	0	9	10	63
*Geobacillus kaustophilus *HTA426	19	28	0	8	15	70
*Geobacillus thermodenitrificans *NG80-2	29	24	0	12	10	75
*Alicyclobacillus acidocaldarius *subsp. acidocaldarius DSM446	29	31	0	9	13	82

^a^GH: Glycoside Hydrolases; ^b^GT: Glycosyl Transferases; ^c^PL: Polysaccharide Lyases; ^d^CE:Carbohydrate Esterases; ^e^CBM: Carbohydrate Binding Modules

Next, we extended the analysis to the various families that constitute each of the five CAZyme classes (Additional File 3). This analysis showed that in comparison with the other *Bacilli *considered in this study, *B. indicus *and *B. firmus *have a high number of CAZymes of the GH13, GT2 and GT4 families and have some CAZymes of families not common in other *Bacilli *(GH2, GH16, GH31, GH35, GH36, GH66, GH84, GH94, GT5, GT27, GT32, CBM4, CBM13, CBM20, CBM41 and CBM56) (Additional File 3).

In addition, we observed the presence in GB1 and HU36 of candidate enzymes for the potential degradation of animal glycans. In particular, GB1 has two candidate β-N-acetylhexosaminidases (GH3, gb1_67550/68320 and gb1_69350/69360) which can target host glycans as well as bacterial cell walls [27], while both GB1 and HU36 have various candidate N-acetylglucosamine deacetylases (CE4, gb1_18820, gb1_34880, gb1_38420, gb1_07440, gb1_46210, gb1_68330, ho_00030, ho_24690, ho_10890, ho_27600, ho_27610) and N-acetylglucosaminidase-6P-deacetylases (CE9, gb1_66390, ho_21030, ho_39690) that can catalyze the elimination of an acetyl group from peptidoglycan N-acetylglucosamine as well as from animal glycan containing O-acetylated sugars (for example, sialic acids) [27].

### The hydrolytic potential of *B. firmus *and *B. indicus *genomes correlates with growth on selected carbohydrates

The CAZy annotation results were compared to the growth profile of *B. firmus *GB1 and *B. indicus *HU36 (Table 2). Overall the growth profiles of both strains on minimal medium supplemented with selected monosaccharides, disaccharides or cellulose correlated with the presence of related CAZymes in their genome (Additional Files 1 and 2). *B. firmus *GB1 was able to grow efficiently in minimal medium supplemented with glucose, fructose, arabinose, mannose, xylose, sucrose or trehalose, as expected by the presence of candidate specific GHs (Additional File 4). Weak growth was observed with galactose, lactose, maltose and cellulose, while growth was not supported only by fucose (Table 2 and Additional File 4). *B. indicus *HU36 was able to grow efficiently in minimal medium supplemented with glucose, fructose, mannose, maltose, sucrose or trehalose, as expected by the presence of candidate specific GHs (Additional File 4). Weak growth was supported by galactose while growth was not observed in the presence of arabinose, fucose, xylose, lactose or cellulose as sole carbon sources in agreement with the absence of candidate specific GHs (Table 2 and Additional File 4).

**Table 2:** Growth and pigment formation in minimal and rich media

	*Bacillus firmus *GB1				*Bacillus indicus *HU36			
	Minimal medium^a^		Rich medium^b^		Minimal medium^a^		Rich medium^b^	
	growth	pigment	growth	pigment	growth	pigment	growth	pigment
NO SUGAR	-	-	+	+	-	-	+	+
Glucose	+	-	+	-	+	-	+	-
Fructose	+	-	+	-	+	-	+	-
Galactose	+/-	-	+	+	+/-	-	+	+
Arabinose	+	-	+	-	-	-	+	+
Mannose	+	-	+	-	+	-	+	-
Fucose	-	-	+	+	-	-	+	+
Xylose	+	-	+	-	-	-	+	+
Lactose	+/-	-	+	+/-	-	-	+	+
Maltose	+/-	-	+	+/-	+	-	+	-
Sucrose	+	-	+	-	+	-	+	-
Trehalose	+	-	+	-	+	-	+	-
Cellulose	+/-	-	+	+/-	-	-	+	+

^a ^M9 minimal medium; ^b^LB rich medium.

We never observed carotenoid formation in solid minimal medium supplemented with any of the carbohydrate analyzed (Table 2). When the same selected carbohydrates were used to supplement rich (LB) medium, growth was always allowed but carotenoid formation was inhibited by all sugars able to support efficient growth as sole carbon source (Table 2). Galactose that, as sole carbon source, weakly supported growth of both *B. firmus *and *B. indicus *did not affect carotenoid synthesis in either organisms (Table 2), while lactose, maltose and cellulose were also able to support a weak growth of *B. firmus *and showed a partial negative effect on carotenoid production (Table 2).

Results of Table 2 are, therefore, suggestive of a catabolite repression-like control on carotenoid biosynthesis in both pigmented *Bacilli*. A negative effect of carbohydrates on carotenoid production was not totally unexpected. Although little is known about the regulation of carotenoid biosynthesis in non-photosynthetic bacteria, it has been previously observed that carotenoid synthesis is repressed by glucose in various species of the genus *Erwinia *[29]. Genes of *Erwinia herbicola *cloned in *Escherichia coli *have been shown to be controlled by a cAMP-dependent catabolite repression mechanism [29]. In the Gram-positive *Myxococcus xanthus *a strong light-dependent induction of carotenoid production only occurs under conditions of carbon starvation [30].

**Figure 1:**
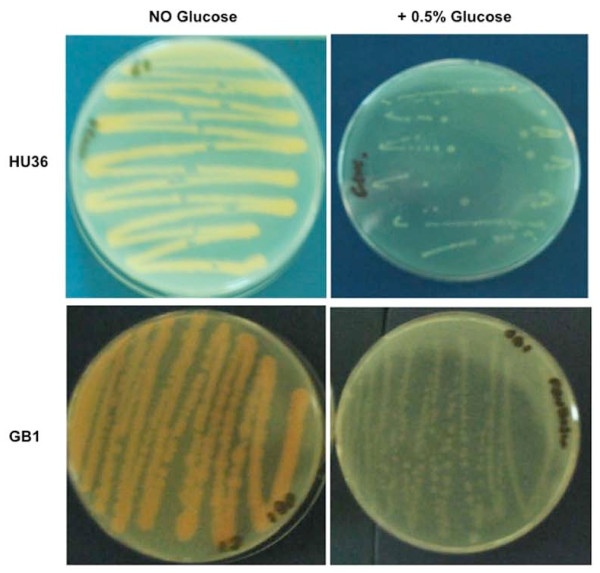
**Growth of the pigmented strains in rich solid medium.** On plates without glucose carotenoid was usually visible after 12-18 hours. Glucose-supplemented plates were left at 37°C for 7 days to check carotenoid production.

**Figure 2:**
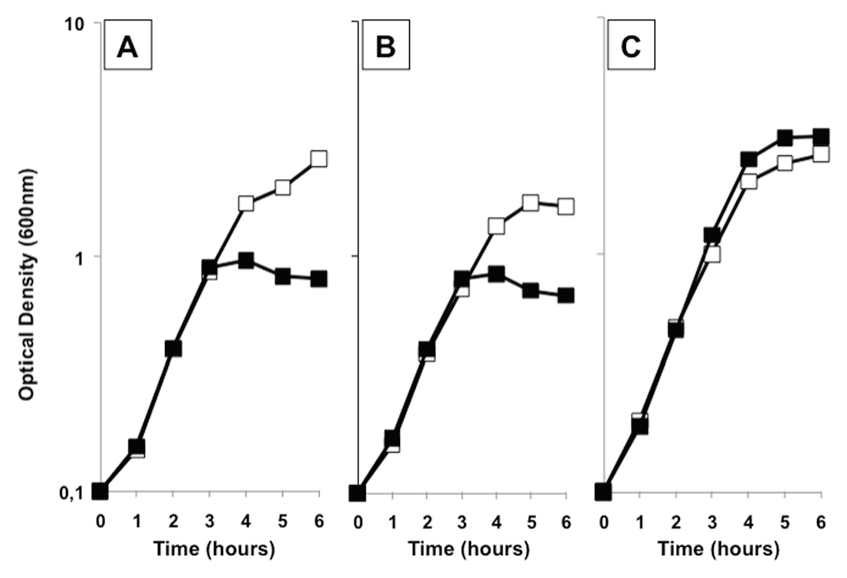
**Growth of the pigmented strains in rich liquid medium. **Growth curve in LB (open squares) and LB supplemented with 0.5% glucose (closed squares) of GB1 (A), HU36 (B) and PY79 (C). Growth was started from overnight liquid cultures in LB diluted at 0.1 OD_600 nm_.

Figure 1 reports the effects of the presence of 0.5% glucose in a rich (LB), solid medium. In addition to repressing carotenoid production, the presence of glucose also appears to reduce the growth of both strains. When 0.5% glucose was added to a liquid, rich (LB) medium, the growth rate of both *B. firmus *GB1 and *B. indicus *HU36 was not affected but cells lysed at the end of the exponential growth phase (Figure 2AB). No differences were observed in either growth or death rates of both strains by decreasing the amount of supplemented glucose to 0.2% or increasing it to 1% (not shown). When the same experiment was performed with an unpigmented strain of *B. subtilis *(PY79) cell death was not observed (Figure 2C). It has been previously reported that during the exponential growth of *B. subtilis*, as much as 17% of the oxygen used for metabolism can be in the form of oxygen radicals and that at the end of the exponential phase of growth, these oxidants may accumulate to toxic levels [31]. Resistance to those oxidants is, then, the result of the induction of the oxidative stress response [31] that in *B. subtilis *occurs because of the concerted action of the superoxide dismutases SodA [32] and the vegetative catalases KatA [31]. As reported in Table 3, the genome of *B. firmus *GB1 encodes for a candidate enzyme with catalase activity but not for a superoxide dismutase while the genome of *B. indicus *HU36 encodes for a candidate superoxide dismutase but not for a catalase. To partially validate the analysis of Table 3 we measured the catalase activity of the two strains and found that while HU36 cells were catalase negative, GB1 cells were positive, although their catalase activity was weaker than that of *B. subtilis *strain PY79 (data not shown). Based on this, we hypothesize that the presence of only a catalase (*B. firmus *GB1) or only a superoxide dismutase (*B. indicus *HU36) does not ensure full protection of the cells against oxygen reactive forms and that production of carotenoids is an essential part of the oxidative stress response in both pigmented *Bacilli*. Therefore, the addition of glucose, repressing carotenoid biosynthesis, would make cells sensitive to the oxygen-derived toxic molecules produced during growth.

**Table 3:** Bioinformatic search for the presence of genes coding for proteins homologous to KatA or SodA of *B.subtilis*

Query	*B. firmus *GB1	*B. indicus *HU36
KatA (NP_388762.2)	contig00442 GENE 1	-
	(90% identity)	
SodA (NP_390381.3)	-	contig00407 GENE 23 (49% identity)

### The hydrolytic potential of *B. firmus *and *B. indicus *genomes correlate with biofilm production

**Figure 3:**
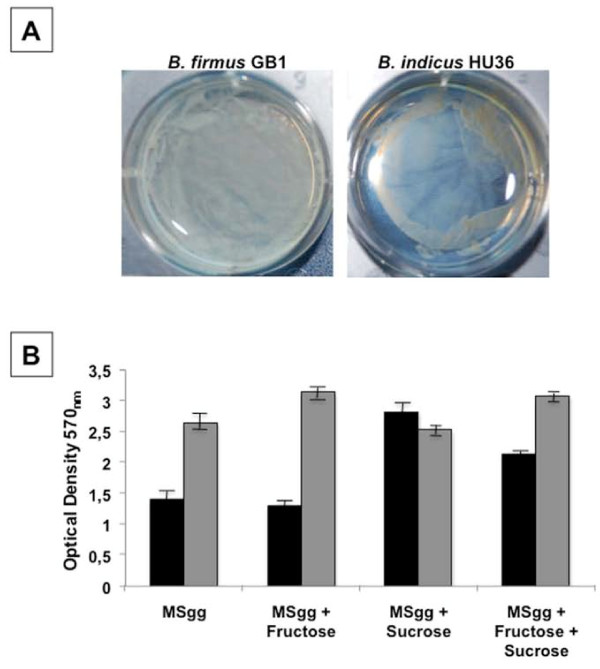
**Biofilm formation. **(A) Biofilm formed by *B. firmus *GB1and *B. indicus *HU36 on a solid MSgg medium. Plates were incubated 4 days at 37°C. Biofilm was visible after about 3 days. (B) Production of biofilm by *B. firmus *GB1 (black bars) and *B. indicus *HU36 (grey bars) in liquid MSgg medium supplemented with 0.5% fructose or 0.5% sucrose or 0.5% fructose and 0.5% sucrose. Data shown are representative of three independent experiments.

Both *B. firmus *GB1 and *B. indicus *HU36 form biofilm in liquid and solid (Figure 3A) media. Wild strains of *B. subtilis*, the model system for spore-formers, form a robust extracellular matrix in which diverse subpopulations of cells involved in sporulation, motility and matrix formation are encased [33]. The extracellular matrix of *B. subtilis *is composed of two proteins, TasA and TapA [34,35] and by an exopolysaccharide (ESP). The most common ESP found in biofilm produced by *B. subtilis *is levan [36] which can be formed by either β-2,6-linked D-fructose units (type I) or a fructose polymer with a glucose residue linked to the terminal fructose by α-glycoside bond (type II). Levan is synthesized outside the cell following the secretion of an extracellular levansucrase (2,6-β-D-fructan-6-β-D-fructosyl-transferase), able to transfer the fructose residue to the acceptor levan when sucrose is used as a substrate [36]. Biofilm formation also requires the action of extracellular levanases (β-D-fructofuranosidase), responsible for levan degradation [36]. Genes for a candidate secreted levansucrase (GH68, ho_13790) and a candidate secreted endo-levanase (GH32, ho_44480) are present in the genome of *B. indicus *HU36 (Additional File 2). The genome of *B. firmus *GB1 did not reveal the presence of enzymes involved in the synthesis of levan but contained the potentials to encode a candidate exo-inulinase (GH32, gb1_42340 and gb1_42350) (Additional File 1). Exo-inulinases are enzymes that hydrolyze terminal, non-reducing 2,1-linked and 2,6-linked β-D-fructofuranose residues in inulin, levan and sucrose releasing β-D-fructose. A candidate fructan exo-inulinase (GH32, ho_44510) is also contained in the genome of *B. indicus *HU36 (Additional File 2).

Based on these observations we suggest that *B. indicus *HU36 produces a levan-based biofilm. Additional experiments will be needed to clarify the nature of the carbohydrate present in the biofilm of *B. firmus *GB1.

In *B. subtilis *levansucrases are induced by sucrose [35] and levanases by low concentrations of fructose [35]. Based on this we analyzed biofilm formation by *B. firmus *GB1 and *B. indicus *HU36 in the presence of sucrose, fructose or both sugars together. As shown in Figure 3B, while in HU36 cells production of the levan-based biofilm was not significantly affected by the presence of fructose, sucrose or both carbohydrates, in GB1 cells biofilm synthesis was about two-fold induced by sucrose and this induction was reduced by the concomitantly presence of the two carbohydrates (Figure 3B). In our standard conditions (MSgg medium) *B. indicus *HU36 (grey bars) was more efficient than *B. firmus *GB1 (black bars) in producing a biofilm.

### The hydrolytic potential of *B. firmus *and *B. indicus *genomes correlate with mucin binding and degradation

Mucins are a family of high molecular weight, heavily glycosylated proteins produced by epithelial cells and forming the viscoelastic gel-like layer that covers the epithelial surfaces in the mammalian GI-tract. The glycosidic part of mucin is formed by linear or branched oligosaccharides that form up to 85% of the molecule by weight. Although chemically and structurally diverse, mucins invariably contain large quantities of galactose, amino sugars, fucose, have strongly polar groups, such as neuraminic (sialic) acids and sulphate at the end of the polysaccharide moiety. Mucins can be degraded by several different hydrolytic enzymes to smaller oligomers, monosaccharides, and amino acids and used as carbon, nitrogen, and energy sources by colonic bacteria. It is commonly accepted that the breakdown of mucins occurs as a cooperative activity in the gut microbiota with different bacteria able to synthesize the variety of hydrolytic enzymes (glycosidases, proteases, peptidases and sulfatases) needed for a complete degradation of mucins [37]. Also important in this regard is the action of deacetylases, enzymes needed to remove O-acetylated sugars that are present at the termini of host glycans to prevent direct cleavage by microbial glycoside hydrolases. Bacteria that have these enzymes therefore produce deacetylated sugars available for them and other components of the microbiota [37].

The CAZy annotation results are consistent with the ability of both pigmented *Bacilli *to adhere and degrade mucin. The *B. firmus *GB1 genome encodes a candidate polypeptide N-acetylgalactosaminyltransferase, belonging to the GT27 family (gb1_47520) and several candidate deacetylases (gb1_18820, gb1_34880, gb1_38420, gb1_07440, gb1_46210) of the CE4 family and a phosphate-deacetylase (gb1_66390) of the CE9 family (Additional file 1). The *B. indicus *HU36 genome encodes a candidate secreted N-acetyl β-glucosaminidase (ho_39680) of the GH84 family, has a Carbohydrate-Binding Module (hu_44470) of the CBM13 family and several candidate deacetylases (ho_00030, ho_24690, ho_10890, ho_27600, ho_27610) of the CE4 family and phosphate-deacetylases (ho_21030, ho_39690) of the CE9 family (Additional file 2). Polypeptide N-acetylgalactosaminyltransferases of family GH27 catalyze the transfer of N-acetylgalactosamine (GalNAc) from the sugar donor UDP-GalNAc to a serine or threonine residue of an acceptor polypeptide and in mammalians are involved in the initial step of O-linked protein glycosylation. The presence of a gene coding for a candidate polypeptide N-acetylgalactosaminyltransferase in the genome of GB1 is a surprising finding and suggests the possibility that GB1 is able to either remodel host glycans or synthesize carbohydrate epitopes mimicking those of the host at the bacterial cell surface.

To experimentally validate those bioinformatic predictions we analyzed the ability of both pigmented *Bacilli *to bind and degrade mucin. Adhesion to mucin was assayed as previously described [38]. In brief, 10^8 ^CFU were incubated in polystyrene tubes pre-treated with mucin, washed extensively and bound bacteria released by treatment with Triton X-100 and plate-counted (Methods). Mucin degradation was assessed by a previously described plate assay [39]. Together with the two pigmented *Bacilli *we analyzed, as control strains, *Lactobacillus rhamnosus *GG (LGG), known to bind and degrade mucin [38] and *L. gasseri *SF1183, previously shown to be unable to degrade mucin [39]. As reported in Table 4*B. firmus *GB1 adhered to mucin with the same efficiency of LGG but was unable to degrade mucin while *B. indicus *HU36 was about 10-fold more efficient than LGG in binding mucin and was also able to efficiently degrade the mammalian glycan.

**Table 4:** Binding to and degradation of mucin by *B.firmus *GB1 and *B. indicus *HU36

Strains	Mucin	
	adhesion^a^	degradation^b^
*Bacillus firmus *GB1	2.5 × 10^3^	**-**
*Bacillus indicus *HU36	30.0 × 10^3^	**++**
*Lactobacillus gasseri *SF1183	ND	**-**
*Lactobacillus rhamnosus *GG	2.0 × 10^3^	**+**

^a ^CFU adhered to plastic wells; ND: not detectable; ^b ^Symbols refers to the size of the degradation halo: - = no degradation halo; + = 1-2 cm; ++ = more than 2 cm.

## Conclusions

The primary result of this work is the annotation of the CAZymes of two carotenoid-producing *Bacilli*. The genome of both the two spore formers contains an elevated number of putative CAZymes, in particular of glycoside hydrolases and carbohydrate binding modules. The total number of CAZymes and the number of putative members of each of the five classes of CAZymes indicated that both *Bacilli *are, and in this respect, similar to the *B. subtilis/B. amyloliquefaciens *group of spore formers and different from thermophilic or facultative alkaliphile strains, presumably living in restrictive environmental niches.

The experimental analysis of the hydrolytic potential of *B. firmus *and *B. indicus *confirmed the genomic analysis and indicated that both *Bacilli *are able to degrade and use as sole carbon source several different carbohydrates. This experimental analysis also allowed us to propose that in both strains a catabolite repression-like mechanism controls carotenoid biosynthesis and that the produced carotenoid is essential to fully protect the growing cells against oxygen reactive forms.

CAZy analyses of the genomes of the two pigmented *Bacilli*, validated by experimental data, also indicated that both strains are able to form biofilm and adhere/degrade mammal mucin. Biofilm formation has been previously associated to a longer persistance in the GI-tract of intestinal *Bacilli *[8], while the ability to bind to and degrade mucin is believed to be a beneficial feature of intestinal bacteria enabling faster mucin turnover and, as a consequence, contributing to the integrity of the intestinal epithelium [40]. The ability to degrade mucin may also be an adaptive advantage for intestinal bacteria, where using mucin as a source of nutrients, can more efficiently colonize the epithelial cell surface underneath the mucus layers [40].

In conclusion, our results suggest that the two pigmented *Bacilli*, isolated from human feces (HU36 [8]) and a human ileal sample (GB1 [6]), are adapted to the intestinal environment and suited to grow and colonize the human gut.

## Methods

### Bacterial growth conditions

*Bacilli *were grown either in LB medium (for 1 l: 10 g Bacto-Tryptone, 5 g Bacto-yeast extract, 10 g NaCl, pH 7.0) or in minimal M9 medium (Na_2_HPO_4 _6 g/l, KH_2_PO_4 _3 g/l, NaCl 0.5 g/l, NH_4_Cl 1 g/l, MgSO_4_.7H_2_O 1 mM, CaCl_2_.2H_2_O 0.1 mM, carbon source 0.2%) in aerobic conditions at 37°C. *Lactobacilli *were grown on deMan, Rogosa and Sharpe (MRS) (Difco) medium in anaerobic condition, obtained by incubating liquid and solid cultures in an anaerobic chamber (Oxoid), at 37°C.

### CAZY annotation

All protein-encoding ORFs from the *B. firmus *GB1 and *B. indicus *HU36 genomes were submitted for analysis using the CAZy annotation pipeline in a two-step procedure of identification and annotation. The identification step of CAZymes followed a procedure previously described [41], where sequences are subject to BLASTp analysis against a library composed of modules derived from CAZy. The positive hits are then subjected to a modular annotation procedure that maps the individual modules against on the peptide using comparisons against libraries of catalytic and carbohydrate models derived from CAZy using BLASTp or Markov models [42]. The results were analyzed for the presence of signal peptide indicating enzyme's secretion and trans membrane domains indicating a membrane anchor, [43]. The functional annotation step involved BlastP comparisons against a library of protein modules derived from the biochemically characterized enzymes found in the Carbohydrate-active enzymes database. The manual comparison with these proteins of known activity yielded three levels of annotation: i) "candidate" activity, ii) "related to" activity and iii) "distantly related to" activity as a function of the distance with functionally characterized enzymes (> 50% identity for candidate, > 30% for related to and less than 30% for distantly related to). In families known to group together enzymes of differing substrate specificity, the "related to" annotation could be upgraded to "candidate" by using a broad activity descriptor, for instance β-glycosidase instead of β-mannosidase.

### Biofilm production

To test biofilm production overnight cultures were used to inoculate liquid MSgg medium (100 mmol l^-1 ^MOPS pH 7.0, 0.5% glycerol, 0.5% glutamate, 5 mm potassium phosphate pH 7.0, 50 μg ml^-1 ^tryptophan, 50 mg ml^-1 ^phenylalanine, 2 mmol l^-1 ^MgCl_2_, 0.7 mmol l^-1 ^CaCl_2_, 50 μmol l^-1 ^FeCl_3_, 50 μmol l^-1 ^MnCl_2_, 2 μmol l^-1 ^thiamine, 1 μmol l^-1 ^ZnCl_2_) [5] and cells grown at 37°C in static conditions for up to 48 h. Cells forming a solid layer at the liquid-air interface were considered as biofilm producers. To quantify biofilm formation, bacteria were grown in MSgg medium at 37°C for 3 days in 6-wells polystyrene microtiter plates. Culture medium was removed and wells washed with phosphate-buffered saline (PBS). The solid biofilm layer was stained for 30 min with two ml 0.1% (wt/vol) crystal violet in an isopropanol-methanol-PBS solution (1:1:18 [vol/vol]). Wells were then washed again with dH_2_O and air-dried (about 30 min). The crystal violet bound to the wells was extracted with 2 ml ethanol-acetone (80:20) and the optical density (OD) of each well was measured at 570 nm.

### Mucin adhesion and degradation assays

Mucin adhesion assays were performed as previously described [Borja et al. 2010]. 100 μl of a mucin (from porcine stomach type III; Sigma-Aldrich) solution in PBS (10 mg/ml) was immobilized on the wells of 96-well polystyrene microtiter plates for one hour at 37°C, followed by overnight incubation at 4°C. Wells were washed twice with 200 μl of PBS and incubated with 20 g/l bovine serum albumin (BSA) (Sigma-Aldrich), for 2 h at 4°C. Non-bound BSA was eliminated by extensive washes with PBS, and 100 μl of bacterial cell suspensions (approximately 10^9 ^CFU/ml), was added to the wells and incubated at 37°C for 1 h. Wells were washed five times with 200 μl of sterile citrate buffer to remove unbound bacteria. Two hundred μl of 0.5% (v/v) Triton X-100 was added to eliminate attached bacteria. The content of each well was thoroughly mixed with a micropipette, and 100 μl of the resulting suspensions plated to obtain the CFU/well. Results are the average of three independent experiments.

Mucin degradation assays were performed as previously reported [Fakhry et al., 2009]. Cells were grown overnight and spotted on Medium B plates: tryptone (Oxoid) 7.5 g/l; casitone (Difco) 7.5 g/l; yeast extract (Oxoid) 3.0 g/l; meat extract (Merck) 5.0 g/l; NaCl (BDH) 5.0 g/l; K_2_HPO-3H_2_O (BDH) 3.0 g/l; KH_2_PO (BDH) 0.5 g/l; MgSO-7H_2_O (BDH) 0.5 g/l; cysteine HCl (Sigma) 0.5 g/l; resazurin (BDH) 0.002. g/l; D-(1)-glucose (BDH) 10 or 30 g/l, purified hog gastric mucin (HGM) 3 g/l and agarose (Sigma) 1.5 g/100 ml. The pH of medium was adjusted to 7.0 with 2 N NaOH. Mucin degradation activity was evaluated by the diameter of the halo observed after plate staining with amido black 0.1% in glacial acetic acid 3.5 M and washing with glacial acetic acid 1.2 M.

## Authors' contributions

All authors read and approved the final version of the paper. NM was the main author of the paper and participated in CAZy annotation and experimental validation. ED contributed to the experimental work on biofilm formation. PMC participated and supervised the CAZy annotation. SMC contributed to the interpretation of the results. BH participated and supervised the CAZy annotation and contributed to the manuscript. ER supervised the experimental work and contributed to the manuscript.

## Supplementary Material

Additional file 1 **Functional CAZY annotation for strain *B. firmus *GB1; excel file; lists all CAZymes found in the genome of *B. firmus *GB1**. Click here for file

Additional file 2 **Functional CAZY annotation for strain *B. indicus *HU36; excel file; lists all CAZymes found in the genome of *B. indicus *HU36**. Click here for file

Additional file 3 **Analysis to the various families that constitute each of the five CAZyme classes; excel file; lists all families of each class of CAZymes found in *B. firmus *GB1 and *B. indicus *HU36 and compare them to those of 14 selected *Bacilli***. Click here for file

Additional file 4 **Candidate glycoside hydrolases active against specific carbohydrates; excel file; lists glycoside hydrolases found in *B. firmus *GB1 and *B. indicus *HU36 grouping them for the specific carbohydrate they hydrolyze**. Click here for file
